# Welfare-Related Behaviors in Chickens: Characterization of Fear and Exploration in Local and Commercial Chicken Strains

**DOI:** 10.3390/ani11030679

**Published:** 2021-03-04

**Authors:** Verena Meuser, Leonie Weinhold, Sonja Hillemacher, Inga Tiemann

**Affiliations:** 1 Institute of Agricultural Engineering, Agricultural Faculty, University of Bonn, 53115 Bonn, Germany; sonja.hillemacher@uni-bonn.de (S.H.); inga.tiemann@uni-bonn.de (I.T.); 2 Institute for Medical Biometry, Informatics and Epidemiology, University Hospital Bonn, 53127 Bonn, Germany; weinhold@imbie.meb.uni-bonn.de; 3 Institute of Animal Science, Agricultural Faculty, University of Bonn, 53115 Bonn, Germany

**Keywords:** local breeds, dual-purpose, fear, exploration, welfare assessment

## Abstract

**Simple Summary:**

Locally adapted chicken strains are frequently discussed as a favorable alternative in poultry production. They might serve as dual-purpose and robust strains that have advantageous behaviors, such as exploration and reduced fear reactions, which might support animal welfare in general. Therefore, it is important to characterize welfare-related behaviors, such as fear and exploration, and to identify these behavioral traits among the diversity of chicken strains. The aim of this study was to characterize fear reactions and exploration based on the avoidance of a novel object and the avoidance of a human in three different chicken strains. To identify interesting alternatives for future poultry production, a layer hybrid (Lohmann Brown), a dual-purpose hybrid (Lohmann Dual) and a locally adapted strain (Rhinelander) were tested for their fear and exploration behavior towards unknown objects and humans. Results showed clear differences in fear and exploration between all three strains, regarding fear of objects and humans. The strain-specific results illustrate the high diversity of behavioral traits. The understanding of behavioral diversity, including fear and exploration, might contribute substantially to future breeding programs, including of commercial strains, and the preservation of local strains in general, which can improve animal welfare on a genetic and individual basis.

**Abstract:**

Fear and exploration are crucial traits determining how animals behave in novel situations, and thus, they influence animal welfare. The aim of this study was the characterization of these behavioral traits among different strains to identify interesting alternatives for future poultry production. Whereas the Novel Object Test (NOT) focuses on fear and exploration of novel objects, the Avoidance Distance Test (ADT) addresses this in the context of humans. Here, a commercial hybrid line, a dual-purpose hybrid and a local adapted strain were tested. For the differences between strains and development of fear, Lohmann Brown (*n* = 714), Lohmann Dual (*n* = 844) and Rhinelander (*n* = 458) were observed weekly until maturity. Results show that fear and exploration towards unknown objects and humans are breed-specific (all *p* < 0.01). Additionally, development of fear in NOT and ADT differed between all three strains (both *p* < 0.01). The expressions of fear of humans or objects should be regarded as characteristics adapted for different husbandry systems and breeding goals, e.g., high exploratory behavior in aviary or high avoidance of predators in free-ranging husbandry or at least a balanced ratio between fear and exploration. Characterization of behavioral traits among different strains, understanding diversity and integrating these behaviors into future breeding and husbandry systems might reflect the need to preserve local strains and the potential to improve animal welfare.

## 1. Introduction

Locally adapted chicken strains are frequently discussed as a favorable alternative in poultry production. Due to constant changes in the poultry industry, such as the ban on caging or the ban on killing male day-old chicks in Germany, new demands are constantly made on the breeding, performance and behavior of hens and roosters. One frequently discussed option is to include locally adapted chicken strains in commercial poultry production, e.g., as dual-purpose chicken or as cross-strains between local and high-performance strains with regional connection. While commercial strains are intensively kept, local native strains are kept under extensive conditions with smaller groups, pens and potentially lower stocking densities, and show better adaptation to local housing conditions [1,2]. Due to reduced performances [1,3,4,5] and missing intensive breeding programs which also can influence behavioral traits, local strains might also preserve advantageous behavioral traits, e.g., reduced fear reactions, increased exploration and better adaptive capabilities. These behavioral traits can favor animal welfare in general [6,7], as animals are often confronted with unknown or threatening stimuli especially in husbandry systems, including free-range. Therefore, local strains could be included in future breeding and should be preserved as animal-genetic resources. Thus, it is important to characterize behavioral traits, e.g., exploration and fear, of these strains. 

Fear can be harmful to health and also affect productivity in husbandry systems [8,9,10,11,12,13]. Fear can be seen as the animals’ reaction to a perceived danger, which, in ideal circumstances, is an adaptive state to protect the animal from psychochemical damage [14]. According to Gray [15], the quality of the fear reaction is influenced by different features, such as novelty and physical characteristics (movement, intensity, duration, suddenness). As an expression of fear, active avoidance can be seen as a general fear response. Exploration is thought to counterbalance fear. Animals explore their environment or novel stimuli and approach them in order to, e.g., find food or water, which makes this exploration behavior essential for survival [16,17]. The information gathered from the animal’s exploratory behavior is also important for foraging or roosting and for identifying and avoiding predators and environmental hazards, and leads to a general exploration of their environment [18,19]. Fear is also strongly negatively correlated to exploration [11,20]. When the animal is confronted with a novel stimulus, the situation will create an approach–avoidance conflict by stimulating the exploration essential to survive (e.g., foraging behavior) as well as the avoidance of potentially threatening situations [16,21]. The response based on this conflict can be defined as fear reaction, when the animal expresses avoidance instead of approaching. An imbalance of fear and exploration can lead to chronic stress if the animals’ coping and adaptation capabilities are exceeded [22]. In order to improve animal welfare, it is necessary to reduce this chronic stress for animals. Especially in husbandry systems, animals are often confronted with novel objects or contact with humans, and are kept in limited space preventing, e.g., an escape response. A decreased reaction of fear towards novel stimuli or humans can be seen as an indicator for a good adaptation to possible changes in their environment [8,14]. A good adaptation to these changes in the environment and a decreased fear reaction, and thus, chronic stress, can not only improve animal welfare but should also be considered as a target for breeding programs [11,23]. 

To implement these described favorable behavioral traits in future breeding and to improve animal welfare, different genetics have to be characterized towards those behavioral traits. To characterize behavioral traits in different genetics, such as fear and exploration, which are often considered in assessment of animal welfare, behavioral tests can be conducted in experimental procedures but also practical on farm [24]. Methods to test the fear behavior are the Novel Object Test (NOT) and the Avoidance Distance Test (ADT), which are conducted to test fear of objects (NOT) and humans (ADT) across a wide range of species, including chickens [24,25,26]. However, in recent literature the characterization of these specific behavioral traits among different strains can rarely be found. A comparison of fear against humans and objects between layer hybrids (Lohman brown plus) and dual-purpose hens (Lohman Dual) was conducted by Giersberg et al. [27]. Here, dual-purpose hens, as a discussed alternative to killing male day-old layer chickens, showed less fear against objects and humans. Furthermore, Giersberg et al. [27] emphasize how little information about differences of these behavioral traits is known among different strains, such as dual-purpose chickens. Behavioral traits, such as fear of humans, are also assessed in different broiler strains [28]. Slow-growing broilers show higher avoidance, and thus, higher fear reactions against humans than fast-growing broilers. However, fear is a strong behavior which is influenced by genetics [29]. Recent studies showed that the genetic selection on low or high fear of humans influence performance parameters such as feed efficiency or number of laid eggs [8]. The selection towards low fear of humans also showed a correlated change of other behavioral traits such as fear of unknown objects or exploration [30,31]. However, including the target of low fear responses into breeding programs might also affects other behavioral traits and performance, or reversely. This leads to the assumption that due to intensive breeding programs of the intensively kept commercial hybrids, their behavioral traits should differ from their extensively kept counterparts. However, when talking about the implementation of these behavioral traits in future breeding, heritability of the traits is an important topic. There are only a few studies about heritability of behavioral traits in poultry, with highly variable results [32]. In the study of Rozempolska-Rucinska et al. [33] heritabilities of fear measured in a NOT differed between tested genetics (0.19/0.08). The heritability of exploration measured in the same NOT differed slightly between genetics (0.17/0.19). Agnvall et al. [30,34] studied heritabilities of fear of humans in red junglefowl and found values of 0.17 for this behavioral trait. These studies show how heritabilities can vary between different strains, but they also show heritabilities for fear of humans and unknown objects that are significantly different from 0.

Genetic comparisons regarding the characterization of fear against novel objects and fear of humans are rarely conducted, especially with local genetics. However, when tested, differences between genetics can be found and characterization of these two responses (object/human) can be important for future breeding. High fear responses to objects indicate low exploration and foraging of the entire environment, which leads to conclusions such as reduced free-range use of the chickens [35] and low adaptation of the animal to the husbandry system. High fear reactions towards humans, on the other hand, may indicate increased defense behavior towards predators, which is especially important in relation to free-ranging [36]. In the future, the development of these two behaviors during rearing will also become a topic of interest, since in the biological rearing of poultry, at least in the EU, the provision of free-range even at the age of a few weeks of life is already being discussed.

Due to the lack of literature regarding characterization of fear and exploration, especially in local chicken breeds, towards an unknown object and a human, the aim of this study was to characterize these behavioral traits based on the avoidance of a novel object (NOT) and the avoidance of a human (ADT) in three different chicken strains from the 1st to the 19th week of life, to identify interesting genetics for future breeding and/or preservation with regards to lower fear reactions. Performance in meat production were also accessed and are reported separately [1]. Thus, we hypothesize that intensively kept hybrids such as Lohmann Brown, dual-purpose hybrids such as Lohman Dual and extensively kept chicken strains such as Rhinelander differ in their fear responses against unknown objects and humans due to their history of intensive breeding and husbandry. Deriving from recent studies, we assume lower fear responses in the dual-purpose breed compared to the laying hybrid and highest fear responses in the local breed, due to the lack of directional breeding programs.

## 2. Materials and Methods 

### 2.1. Ethics Statement

Animals were kept according to the German Order on the Protection of Animals and the Keeping of Production Animals (25, last revision 2017). In this study, any procedures for handling animals followed the instructions of the Welfare Quality^®^ Assessment Protocol for Poultry [24], especially the Appropriate Behavior and Positive Emotional State (see 6.1.4.3, p. 76 and 6.1.4.4, p. 77) part. The Campus Frankenforst of the Faculty of Agriculture, University of Bonn was approved as the trial farm (39600305-547/17). 

### 2.2. Animals and Housing

In this study, animals of different strains were tested for animal-based behavioral welfare parameters. Therefore, in November, animals of the dual-purpose hybrid line Lohmann Dual (LD), layer hybrid line Lohmann Brown (LB) and the local dual-purpose strain Rhinelander (RH), which is indigenous to Germany and named on the red list to be endangered, were reared separated from each other in mixed-sex groups. A total of 844 LD chicks and 458 RH chicks were hatched in the Poultry Research Center in Rhein-Kreis Neuss, Germany, and 714 LB chicks were hatched on the same date in the hatchery (Geflügelzuchtbetriebe Gudendorf-Ankum GmbH Co, Ankum, Germany). All chicks were transferred to and raised in the barns of the Campus Frankenforst of the Faculty of Agriculture, University of Bonn (Königswinter, Germany). From the 1st until the 10th week of life, experimental groups consisted of all animals that hatched. Starting at the 11th week of life, 100 cockerels for each strain remained for the behavioral experiments whereas all other cockerels were slaughtered, and all pullets transferred to laying barns. Thus, from the 10th to the 19th week, experimental groups consisted of 100 cockerels per strain until their slaughtering in the 20th week of life. 

The mixed-sex groups of LB, LD and RH were raised and kept under conditions of conventional free-range husbandry. There were three identical pens. Each strain was raised and kept separately in one pen. The pens had a uniform size of 33.82 m^2^ (7.60 m × 4.45 m) and were equipped with three daylight windows. Furthermore, the animals had free-range access outside to 210 m^2^. From the 1st to the 10th week of life, mixed-sex groups (male–female ratio: 1:1.4 in LB, 1:1.5 in LD, 1:1.35 in RH) were raised in these pens as experimental groups; cockerels were separated after the 10th week until the 20th week (*n* = 100), as is done in practice for rearing brother cocks in husbandry systems, and kept as experimental group. Stocking densities (animal/m^2^) were 21.08 (LB), 24.95 (LD) and 13.54 (RH) until week 10, calculated as the average stocking densities from the 1st to the 10th week and 2.70 (LB), 2.96 (LD) and 2.96 (RH), also calculated as average stocking densities, from the 11th to the 19th week. The floor of the pen was littered with woodshavings (Allspan^®^ classic, Allspan Spanverarbeitung GmbH, Karlsruhe, Germany), which was periodically re-scattered or cleaned. During the first two weeks of life, the pen area was reduced to approximately 20 m^2^ by cardboard partitions in order to keep the chicks in the area of the installed heat lamps (Artas type 70,230 Sn, 175 Watt). Feed and water dispensers covered at least 0.66 cm per 1000 g live weight. Two wooden frames served as perches (three perches at 3.50 m). The manual ventilation system could be switched on when needed. Food, water and the health of the animals were checked twice a day. Manipulatable material was available such as straw and apples on chains. Free-range was accessible starting on week 8 of life. The nutritional management followed a conventional feeding plan for broilers and was identical for all strains. For the first two weeks, the animals were fed Landkornstarter pellets (21.5% Crude protein, 0.6% Methionine, 0.9% Calcium, 0.6% Phosphorus, 12.40/kg MJ ME, Coccidiostat), followed by Landkornmast (21.0% Crude protein, 0.5% Methionine, 0.8% Calcium, 0.6% Phosphorus, 12.40/kg MJ ME, Coccidiostat) until one week before slaughtering, when they were fed Landkornendmast (20.0% Crude protein, 0.5% Methionine, 0.8% Calcium, 0.5% Phosphorus, 12.40/kg MJ ME). Breeding groups were fed All-mash L (16.5% Crude protein, 0.35% Methionine, 3.6% Calcium, 0.5% Phosphorus, 11.2/kg MJ ME; all Deutsche Tiernahrung Cremer GmbH & Co KG, Düsseldorf, Germany). Animals were fed for ad libitum intake regarding food, water and grit. Chickens followed a vaccination plan issued by the veterinarian in charge of the flock (including Marek’s disease, Newcastle disease, infectious bronchitis, laryngotracheitis, coccidiosis and Gumboro). 

### 2.3. Experimental Procedure

Due to the great variability of the methods of novel object and human-animal relationship tests, we decided to follow the standardized and established guidelines of the Welfare Quality^®^ Assessment Protocol [24], and thus, we also adopted the naming of the behavioral tests NOT and ADT [24]. Tests were conducted from the 1st to the 19th week of life.

#### 2.3.1. Novel Object Test

For testing, a novel object was placed in the pen of LB, LD and RH chickens. In this study, four different 3 cm thick colored wooden shapes with a diameter of 15 cm (yellow star, green square, green triangle and red circle) were used. The test was carried out every seven days in the morning, starting at the seventh day of life, from the 1st to the 19th week of life. The objects were used in a weekly alternating rhythm. After entering the pen, four observation points in the pen were identified, which represented the distribution of the flock. Observation points were the same in every week and for every strain. In the beginning, the experimenter waited five minutes until animals were undisturbed again. Afterwards, one of the novel objects was laid on the observation point, and the experimenter went back two steps. For two minutes and in a 10-s interval, the number of animals close to the object was documented. During this period, every animal in the pen had the opportunity to react to the object. The scoring radius was equal to the length of one animal, which corresponded to the current size of the animals at each point of time. An animal was scored as soon as one part of the body, for instance the head, undercut the distance of one animal length to the object. Thus, data at 12 intervals after placement of the object (from 10 sec to 2 min) were collected. Afterwards, the experimenter went to the next point and proceeded testing. 

#### 2.3.2. Avoidance Distance Test

The test was carried out once a week right after the NOT with LB, LD and RH chickens. For testing, the experimenter approached a group of at least three animals in the pen before squatting for 10 s. After this time, the number of animals that undercut the distance of one arm’s length to the experimenter (approximately 1 m) was documented. Here, again, every animal in the pen had the opportunity to react to the human. Thus, the measurements referred to the individual animal level, although they were carried out in the pen. This procedure was performed at 21 selected places, named observation points, in each of the pens of the RH, LD and LB chickens. Distribution of observation points was identical between the strains. 

### 2.4. Levels of Analysis within the Study 

This study examines fear and exploration on two different levels (development, genetic), which will be outlined in the following subsections.

#### 2.4.1. Development Level

First, to analyze the development change in fear and exploration behavior from the 1st to the 19th week of life, the NOT and ADT results of LB, LD and RH were analyzed. Because of the change in sex ratios of the groups at the 10th week of life (mixed sex to all-male groups), the analysis was stratified into two blocks of the different age classes covering weeks 1–10 and 11–19. 

#### 2.4.2. Genetic Level

For the strain level, the NOT and ADT results of LD, LB and RH were compared. Because of the change in sex ratios of the groups at the 10th week of life (mixed sex to all-male groups), the analysis here was stratified into two blocks covering weeks 1–10 and 11–19. The NOT and ADT results from weeks 1–10 were analyzed for genetic differences, and separately from this, the results from weeks 11–19 were analyzed for genetic differences. 

### 2.5. Statistical Analysis

#### 2.5.1. Development Level

The development of the ADT and NOT from the 1st to the 19th week of life of LB, LD and RH was analyzed using Poisson generalized mixed models. To compare the development of NOT and ADT results in the 1st to the 10th week of life and the 11th to the 19th week of life, respectively, Poisson regression models were calculated. To investigate the breed-specific changes over time, the models included the variables breed, week of life and the interaction between strain and week of life as covariates. Additionally, stocking density and number of animals were considered as confounder variables in the model. Furthermore, to account for possible dependencies between observation points over the weeks of life, the variable observation point was included as a random effect.

#### 2.5.2. Genetic Level

To compare the results of NOT and ADT across strains of the 1st to the 10th week of life and the 11th to the 19th week of life, respectively, Poisson regression models were calculated. We stratified the analyses by weeks of life (weeks 1–10 and 11–19), as in week 10, the group constellation of all strains changed from mixed sex to all-male cockerels. For all models, stocking density (scaled prior to analysis) was considered as a confounder variable.

All statistical models were calculated on the raw dataset. However, to better visualize the high influence of the stocking density, which was found in the statistical analysis, the visualized values in the Figures of the development level of ADT and NOT were modified for the stocking density. The modified measurements were calculated by:

(1) $${\mathrm{NOT}}_{\mathrm{mod}}/{\mathrm{ADT}}_{\mathrm{mod}}=\frac{\mathrm{number}\;\mathrm{of}\;\mathrm{animals}\;\mathrm{around}\;\mathrm{object}/\mathrm{human}}{\mathrm{number}\;\mathrm{of}\;\mathrm{animals}\left[n\right]*\mathrm{pen}\;\mathrm{size}{\left[{\mathrm{qm}}^{2}\right]}^{-1}}$$

where the number of animals around the object/human was divided by the stocking density. The calculation was created by the authors themselves. Modified values were not considered for the statistical models, but only to visualize the influence of the stocking density. 

The statistical analysis was carried out with the SPSS^®^ Statistics 27 program (IBM Corporation, Armonk, NY, USA) and R Software for Statistical Computing, version 3.6.1 Version 1.1.9.0, (R Foundation for Statistical Computing, Vienna, Austria) [37]. Graphical representation of the results was conducted with SigmaPlot 13.0 (Systat Software Inc., Chicago, IL, USA). The significance level α was set at *p* ≤ 0.05 and indicated as *, *p* ≤ 0.01 is indicated as ** and *p* ≤ 0.001 as ***.

## 3. Results

### 3.1. Development Level

**Figure 1:**
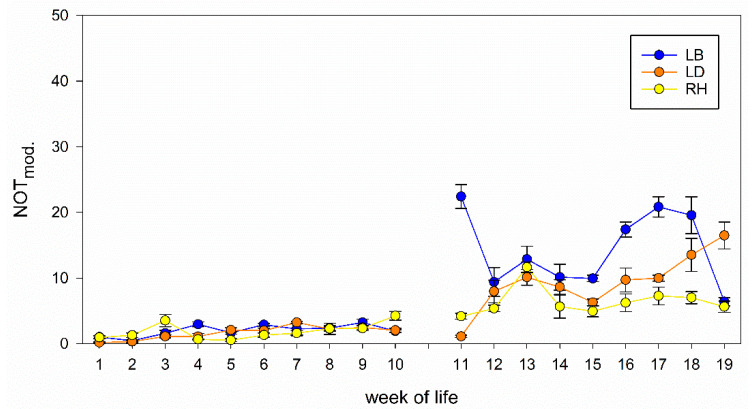
Development of the NOT results. Modified values of NOT (NOT_mod._) for each of the 19 weeks of life of LB, LD and RH. Data are shown that already take the stocking density into account for reasons of illustration.

The analysis of the NOT results showed differences between the strains, related to the development of the results in both the 1st to the 10th and the 11th to the 19th week of life. A strong interaction between week of life and strains was found in both age classes, meaning that the development of fear of the chickens differ between the strains both age classes (both *p* < 0.001, Table 1). Additionally, a significant effect of stocking density on NOT results could be found from week 1 to 10 (*p* < 0.001) and 11 to 19 (*p* = 0.018). Figure 1 shows the results modified for stocking density and number of animals. Here, all strains show constantly low values from the 1st to the 10th week. After changing the sex ratios and reducing the stocking density to a number of 100 cockerels in week 10, the modified values, especially of LB, increase in the 11th week compared to the 1st to the 10th week. Modified values of LD start increasing in the 12th week and constantly increase until the 19th week. Modified values of RH start increasing in the 10th week and do not reach the same level as the other two strains by end of observation period. Modified values of RH are the lowest of all strains from the 14th to the 19th week. 

**Figure 2:**
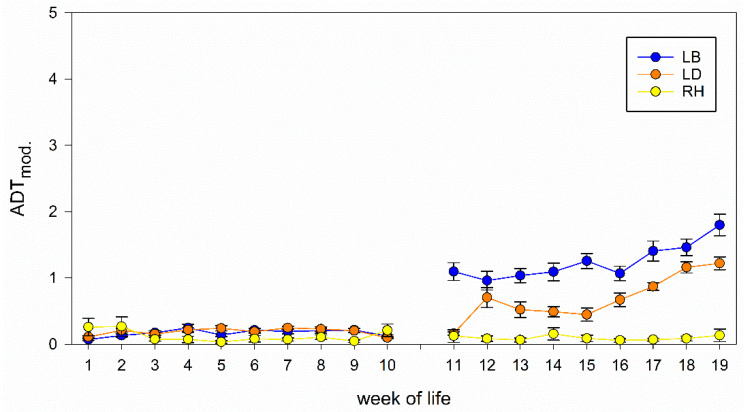
Development of ADT results. Modified values of ADT (ADT_mod_.) for each of the 19 weeks of life of LB, LD and RH. Data are shown that already take the stocking density into account for reasons of illustration.

The analysis of the ADT results also showed a strong interaction between week of life and strain, meaning that the development of fear of the chicken differ between the strains from week 1 to 10 (*p* < 0.001) and week 11 to 19 (*p* = 0.015, Table 2). Furthermore, a significant effect of stocking density on results of ADT was found from week 1 to 10 (*p* < 0.001). The modified values for RH stay constant over the 19 weeks of life, whereas modified values of LB and LD start to increase strongly in the 11th and the 12th week, respectively (Figure 2). The values increase from the 11th to the 19th week (LB) and from the 12th to the 19th week (LD), respectively. 

### 3.2. Genetic Level

**Figure 3:**
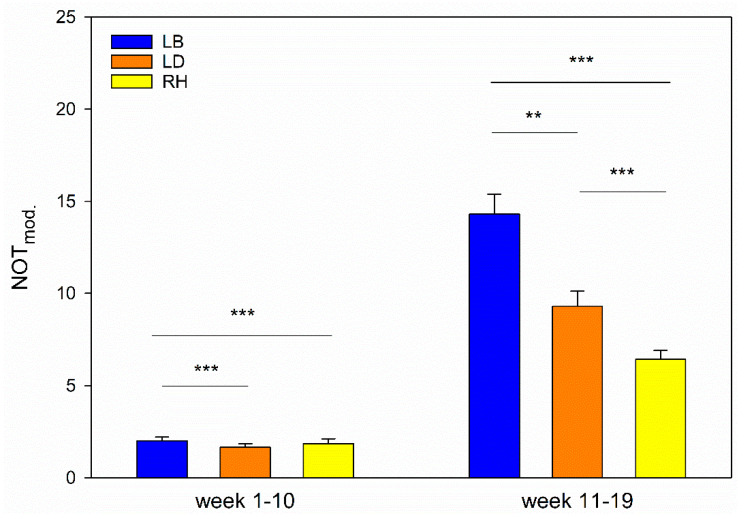
Comparison of strains. Modified NOT values (NOT_mod._) of LB, LD and RH stratified by weeks of life (1–10 and 11–19). Standard errors and significance levels are given (** *p* < 0.01; *** *p* < 0.001). Data are shown that already take the stocking density into account for reasons of illustration.

The regression models investigating strain differences in NOT revealed several pairwise differences. The results of pairwise comparisons of the strains are given in Table 3 (weeks 1–10) and 4 (weeks 11–19); the modified values of NOT are presented in Figure 3. 

Mixed-sex groups of the three strains show significant differences in reactions towards the unknown objects in NOT. Firstly, LB showed highest values in NOT, meaning that a high number of animals undercut the distance to the unknown object (both *p* < 0.001). Table 4 shows ratios of 0.7 between RH and LB and LD and LB, respectively. Compared to the number of LB animals that undercut the distance to the object, RH and LD reached only 70% (ratio = 0.7) of the measured LB value. However, values of LD and RH do not differ (ratio = 1.0, *p* = 1.000). Thus, in first 10 weeks of life in the mixed-sex groups, LB showed lowest fear responses, while LD and RH showed comparable and higher fear responses towards unknown objects. These results are also reflected by the mean values given in Table 3.

Similarly, the all-male groups differed in their responses in NOT from the 11th to the 19th week. Identical to the mixed-sex groups, LB showed the highest measurements of animals undercutting the distance to the unknown object. Compared to LB, the pairwise comparisons showed a ratio of 0.9 for LD (*p* = 0.003) as well as a ratio of 0.7 for RH (*p* < 0.001). Contrary to the mixed-sex groups, LD and RH also differed significantly (*p* < 0.001). Therefore, more LD cockerels undercut the distance to the unknown object (ratio = 1.2). Thus, LB cockerels showed the lowest fear responses, while LD showed intermediate and RH highest fear responses in week 11 to 19. These results are also reflected by the mean values given in Table 4.

**Figure 4:**
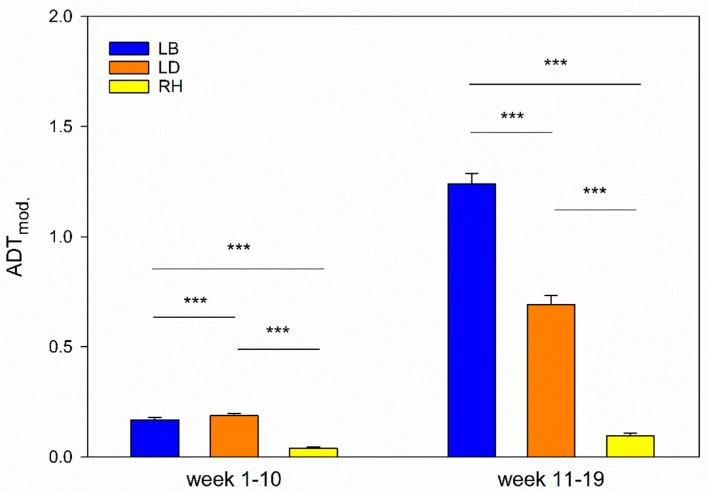
Comparison of strains. Modified ADT values (ADT_mod_.) of LB, LD and RH stratified by weeks of life (1–10 and 11–19). Standard errors and significance levels are given (*** *p* < 0.001). Data are shown that already take the stocking density into account for reasons of illustration.

The regression models investigating strain differences in ADT revealed several pairwise differences. The results of pairwise comparisons of the strains are given in Table 5 (weeks 1–10) and Table 6 (weeks 11–19); the modified values are presented in Figure 4.

Mixed-sex groups of the three strains showed differences in reactions towards humans in the ADT from the 1st to the 10th week. Here, LD showed the highest values, i.e., compared to the other strains, most animals undercut the distance to the human in the pen (both *p* < 0.001). Thereby, compared to LD, pairwise comparisons yielded a ratio of 1.2 for LB and 0.1 for RH (both *p* < 0.001). Additionally, comparisons showed significant differences between LD and RH (*p* < 0.001), with a ratio of 8.2. However, if we consider the average values, we see that the actual difference between LD and LB is rather small. Thus, in the mixed-sex groups, LD and LB showed lowest fear responses and RH showed the highest fear responses toward humans in week 1 to 10.

Similar to the results of the mixed-sex groups from the 1st to the 10th week of life, all strains of the all-male groups differ from each other from the 11th to the 19th week. Here, LB can be identified as the strain with the highest values of animals which undercut the distance to the human (both *p* < 0.001). Therefore, pairwise comparisons yielded ratios of 0.6 when LB and LD are compared, whereas the ratio between RH and LB stands at 0.1. Pairwise comparisons, again, found differences between LD and RH (*p* < 0.001), showing a ratio of 5.5. Additionally, the all-male group from the 11th to the 19th week of LB showed the lowest fear responses towards humans, whereas LD showed intermediate and RH the highest fear responses. These results are also reflected by the mean values given in Table 6.

## 4. Discussion

The characterization of behavioral traits is an important topic, especially as locally adapted and/or dual-purpose strains are discussed as favorable alternatives in poultry production. These locally adapted and dual-purpose strains might preserve advantageous behavioral traits which favor animal welfare. Fear of objects and humans are important behavioral traits, as they affect animal welfare in husbandry systems [22,38]. Thus, the characterization of different strains regarding to these two behavioral traits could bring important beneficial information for future breeding, animal welfare and preservation of strains and behavioral traits. Nevertheless, assessment of animal’s reactions towards unknown objects and humans and the direct comparison between strains can rarely be found in recent literature. Therefore, in this study, fear reactions against unknown objects and humans were assessed in a layer hybrid line (LB), a dual-purpose line (LD) and a locally adapted chicken strain (RH) and compared with each other in the following. 

To consider the effect of mixed-sex rearing, as common for dual-purpose strains and local genetics, the comparison of strains was separated between the mixed-sex groups (weeks 1–10 of life) and the male groups (weeks 11–19 of life). However, the stocking density was also considered, as the calculated model was modified for stocking density. Both in the mixed-sex and the male groups, LB always showed lowest fear responses towards objects and humans with one exception in the NOT of the mixed-sex groups. Here, the mean values of LB and LD were almost identical. However, in all other comparisons, LB always showed the lowest fear responses. This means, compared to the other breeds, more LB animals approached the object/human instead of avoiding it. In contrast, fewer LD approached the object/human and least RH approached the object/human. One exception are the results of the mixed-sex groups of LD and RH, which show similar reactions in NOT. Nevertheless, these results clearly show differences in strains. Thus, when the three strains were confronted with a novel stimulus, the previously described approach–avoidance conflict [16,17,18,19] showed different reactions in each strain. While more LB approached, expressing exploration instead of avoidance [14,16,17,21], fewer LD expressed exploration and more expressed fear. However, in comparison, RH expressed the least exploration and the most fear. Giersberg et al. [27] found contrasting results. In their study, NOT and ADT were also conducted with a layer hybrid (LB+) and a dual-purpose hybrid (LD). However, dual-purpose hybrids were less fearful than the layer hybrids. These differences might be caused by the different ages of the tested animals. While the animals in this study were tested in the first 19 weeks of life, Giersberg et al. [27] tested animals from week 21 to 69 [39]. 

Nonetheless, in our study, the additional comparison of the commercial strains to a locally adapted strain indicates a highly significant difference in the behavioral traits. More LB approached the objects and humans, which indicates a pronounced exploration and foraging behavior [16,17,35] and a less pronounced avoidance of potential predators [36]. LD show intermediate reactions in both, but RH reactions indicate a less pronounced exploration behavior, but a pronounced avoidance of potential predators. Exploration, as a behavior essential to survive, can be seen as a positive behavioral trait, which is pronounced in LB. However, avoiding potential predators, e.g., in free ranging of chickens, is also a behavior essential to survive. Thus, the pronounced avoidance of the human in RH does not necessarily have to be considered as a negative reaction. However, if the fear of the animal becomes permanent, it leads to distress, which must be prevented to increase animal welfare. This leads to the assumption that a balanced relation of exploration and fear of unknown objects and humans is needed, which can be achieved by implementing animals such as LB, LD or RH with appropriate behavioral traits into future breeding. 

Since in the biological rearing of poultry in the EU, the provision of free-range even at the age of a few weeks of life is already being discussed, even the development of fear in the first weeks of life before maturity became a relevant topic in research. In recent literature, the development of fear of unknown objects and humans in the first weeks of life also can rarely be found [40]. Thus far, no other studies were found repeating the ADT and NOT weekly until the 19th week of life. However, Adler et al. [40] repeated the two behavioral tests weekly until day 28 with broiler chickens and found decreasing fear responses over time. 

In this study, differences between the strains were found in the development of fear in the first 20 weeks of life, analyzed separately from week 1 to 10 and 11 to 19. Results showed strong interactions between week of life and strain, meaning the development of NOT and ADT differs between strains in both age groups. Thus, this interaction exists despite the large differences in stocking density and sex ratio between the two age classes. This would strengthen the assumption that the differences in the development of fear in NOT and ADT are foremost strain specific. The (towards stocking density) modified values revealed a change of fear behavior after the 10th week of life. In the 10th week, the number of animals was reduced to only 100 cockerels for each genetic strain (LB, LD and RH), and thus, stocking density decreased. Additionally, the sex ratio changed from mixed sex to male groups. Nevertheless, modified NOT values show strong decreasing fear responses toward the novel object for LB after 10th week and also for LD and RH after the 11th week. Modified ADT values show decreasing avoidance, and thus, fear responses toward the human for LB after the 10th week and LD after the 11th week. However, fear responses towards humans did not change in RH. Due to the constant low response level of RH in ADT, also after reducing stocking density and sex ratio, it can be assumed that an impact of stocking density or sex on fear of humans cannot be found, though a fear of unknown objects is present. Fear responses in RH might, therefore, be referential with a higher impact of humans compared to objects on fear. On the contrary, commercially used strains LB and LD show decreased avoidance of objects and humans after reducing stocking density and changing the sex ratio. This might be due to their history of intensive breeding and selection programs. Intensive breeding and handling as well as high performances might have favored low fear levels [8]. As in the comparison of strains, in the development of fear, RH show a pronounced avoidance against potential predators. Unknown animals and potential predators are avoided, which is also a behavior essential to survival. Thus, it should be considered that strongly decreased fear of humans in LD and LB also can be a negative impact when it comes to avoidance of potential predators in free ranged chickens [36]. Therefore, a good balance between fear and exploration is a major concern for future breeding. Here, again, the diversity of the two behavioral traits between the strains is apparent, this time regarding the development of fear and exploration. By the exact characterization, humans do not only have the possibility to influence the animals by selective breeding, but also to adapt husbandry systems to the selected animals regarding their behavior, thereby reducing fear and the resulting stress for the animals and finally to increase animal welfare. 

The influence of stocking density on fear, welfare and behavior in general is a current and frequently discussed topic [41,42,43]. Different studies already show a positive impact of reduced stocking density on locomotion and foraging activity [44], lameness [45] and leg health [46]. Our results similarly indicate decreasing fear reactions for all strains in NOT and for LB and LD in ADT when stocking density is reduced. These results suggest that fear can be reduced and exploration can be increased, and therefore, the welfare of the animals can be improved when lower stocking densities in husbandry environments are applied. Accordingly, a high stocking density might trigger fear behavior of the animals. However, it is important to consider that the effects of strain and stocking density are confounded, meaning that although both factors were included separately in the analyses, we cannot clearly state if the changes and differences presented in results of NOT and ADT are caused by strain or stocking density. Future studies should address the strain-specific impact of stocking densities on the behavior of chickens as, e.g., local strains might not be adapted to high animal numbers and might, therefore, react differently to intensive housing systems.

Indeed, not only does the change in stocking density at 10 weeks of life represent a significant influencing factor, but also the general difference in stocking density between the commercial strains and the local strain RH, which affects the development of diverse behavioral and performance parameters from the first day of life [41,42,43,44,45,46,47]. Nevertheless, Eugen et al. [47] showed a threefold in- or decrease of the stocking density results in higher fear and anxious behavior. Thus, the pronounced or less pronounced fear reactions of all three strains can be influenced by the stocking density during rearing. However, the intensive breeding programs and husbandry of the commercial hybrids could have led to a tolerance of higher stocking densities, compared to locally adapted strains and their extensive husbandry. These locally adapted strains, thus, might have other requirements to their environment as compared to the hybrid lines. Kozak et al. [33] even concluded that each individual laying line has different environmental requirements. This could also apply to local genetics and influence the best possible stocking density for the strain, as one requirement is the space to exhibit behavioral traits such as fear reactions [33]. Due to the influence of stocking densities on fear, this factor should be considered in future research, e.g., by testing the behaviors of strains under different stocking densities. However, in this study, the differences among the strains were clearly noticeable, even if their stocking density slightly differed from beginning, such as LB and LD. Thus, we assume that differences among the strains are influenced but not fully explainable by the difference in stocking density. 

Extensively kept local chicken strains might favor predator/human avoidance and explore novel objects, whereas intensively commercial chicken strains benefit from less fear towards humans and objects in their husbandry systems. Due to changing societal interests to keep more chickens in free-range, it has to be considered that commercial origins are not adapted to this. Animal genetic resources might offer starting points for future breeding programs to enable balanced fear and exploration responses to new stimuli in all animals in their husbandry systems, and thus, promote positive affective states and general animal welfare. 

## 5. Conclusions

The results of this study show a high behavioral diversity towards objects and humans between the tested strains and, therefore, support our hypothesis that intensively kept hybrids such as Lohmann Brown, dual-purpose hybrids such as Lohman Dual and extensively kept local chicken strains such as Rhinelander differ in their fear responses against novel stimuli. It can be assumed that these differences appear due to their genetics, different breeding histories and reduced performances of local strains. The characterizations of the strains in this study not only show a great diversity of the behaviors investigated, they also confirm that further studies on the characterization of behavioral traits of different strains are of great importance for future breeding, the preservation of local strains and the general improvement of animal welfare. The behavioral traits clearly differ between strains, although responses are not clearly categorizable into positive or negative. The expressions of fear of humans or objects should be regarded as characteristics adapted for different husbandry systems and breeding goals, e.g., high exploratory behavior in aviary or high avoidance of predators in free-ranging husbandry, or at least a balanced ratio between fear and exploration. The results of this study reflect the diversity of behaviors and provide a good basement for further characterization of different strains to improve animal welfare, support future breeding and preserve local breeds. 

## Figures and Tables

**Table 1:** Development of NOT results for LD, LB and RH. The 95% confidence intervals are shown for the exponentiated coefficients.

Variables	Exp(estimate)	95%-Confidence Interval		*p*-Value
		Lower	Upper	
**weeks 1–10**				
(Intercept)	0.22	0.07	0.63	
Week of life	2.79	2.42	3.22	<0.001
Genotype				<0.001
Stocking density (kg/m^2^)	0.21	0.17	0.26	<0.001
Number of animals	1.78	1.04	3.04	0.035
Week of life: Genotype				<0.001
Week of life: Genotype RH	0.51	0.46	0.57	
Week of life: Genotype LB	0.66	0.61	0.71	
**weeks 11–19**				
(Intercept)	14.68	6.80	31.29	
Week of life	1.12	1.09	1.16	0.0011
Genotype				<0.001
Stocking density (kg/m^2^)	0.59	0.39	0.92	0.018
Number of animals	2.76	1.14	7.11	0.023
Week of life: Genotype				<0.001
Week of life: Genotype RH	0.90	0.86	0.94	
Week of life: Genotype LB	0.88	0.85	0.91	

Notes: Results of the Poisson generalized mixed model for NOT included the variables breed/week of life/interaction between strain and week of life as covariates. The model was modified for stocking density/number of animals/the variable observation point, which were included as random effects.

**Table 2:** Development of ADT results for LD, LB and RH. The 95% confidence intervals are shown for the exponentiated coefficients.

Variables	Exp(estimate)	95%-Confidence Interval		*p*-Value
		Lower	Upper	
**weeks 1–10**				
(Intercept)	0.49	0.13	1.63	
Week of life	1.76	1.50	2.07	0.0012
Genotype				<0.001
Stocking density (kg/m^2^)	0.32	0.24	0.43	<0.001
Number of animals	1.04	1.65	2.16	0.914
Week of life: Genotype				<0.001
Week of life: Genotype RH	0.57	0.49	0.65	
Week of life: Genotype LB	0.85	0.78	0.93	
**weeks 11–19**				
(Intercept)	1.35	0.14	10.12	
Week of life	1.17	1.06	1.11	<0.001
Genotype				<0.001
Stocking density (kg/m^2^)	0.52	0.24	1.20	0.378
Number of animals	6.40	0.40	98.81	0.236
Week of life: Genotype				0.015
Week of life: Genotype RH	0.94	0.85	1.07	
Week of life: Genotype LB	0.92	0.88	0.97	

Notes: Results of the Poisson generalized mixed model for NOT included the variables breed/week of life/interaction between strain and week of life as covariates. The model was modified for stocking density/number of animals/the variable observation point, which were included as random effects.

**Table 3:** Genotype differences in NOT in the first 10 weeks of life. Results of the pairwise comparisons of the NOT results regarding to the different strains.

**Breeds**	**Ratio**	**SE**	***p*-Values**
LD–RH	1.0	0.05	1.000
LD–LB	0.7	0.03	<0.001
RH–LB	0.7	0.03	<0.001
	**Mean**	**SD**	**SE**
LB	2.03	1.12	0.18
LD	1.68	1.06	0.17
RH	1.87	1.49	0.24

Notes: Additionally, means, standard deviation (SD) and standard errors (SE) of the modified ADT values for each strain are presented.

**Table 4:** Genotype differences in NOT in the 11th to the 19th week of life. Results of the pairwise comparisons of the NOT results regarding to the different strains.

**Breeds**	**Ratio**	**SE**	***p*-Values**
LD–RH	1.2	0.06	<0.001
LD–LB	0.9	0.04	0.003
RH–LB	0.7	0.03	<0.001
	**Mean**	**SD**	**SE**
LB	14.32	6.38	1.06
LD	9.31	4.90	0.82
RH	6.44	2.80	0.47

Notes: Additionally, means, standard deviation (SD) and standard errors (SE) of the modified ADT values for each strain are presented.

**Table 5:** Genotype differences in ADT in the first 10 weeks of life. Results of the pairwise comparisons of ADT results regarding the different strains.

**Breeds**	**Ratio**	**SE**	***p*-Values**
LD–RH	8.2	0.85	<0.001
LD–LB	1.2	0.06	<0.001
RH–LB	0.1	0.02	<0.001
	**Mean**	**SD**	**SE**
LB	0.17	0.15	0.01
LD	0.19	0.14	0.01
RH	0.01	0.07	0.01

Notes: Additionally, means, standard deviation (SD) and standard errors (SE) of the modified ADT values for each strain are presented.

**Table 6:** Genotype differences in ADT in week of life 11 to 19. Results of the pairwise comparisons of ADT results regarding to the different strains.

**Breeds**	**Ratio**	**SE**	***p*-Values**
LD–RH	5.5	0.71	<0.001
LD–LB	0.6	0.05	<0.001
RH–LB	0.1	0.01	<0.001
	**Mean**	**SD**	**SE**
LB	1.24	0.64	0.05
LD	0.69	0.54	0.04
RH	0.10	0.16	0.01

Notes: Additionally, means, standard deviation (SD) and standard errors (SE) of the modified ADT values for each strain are presented.

## Data Availability

The data presented in this study are available on request from the corresponding author. The data are not publicly available due to complexity and extent of the data source, whereas outcome of all statistical models are presented in the tables of this manuscript.
